# Acyl Ghrelin Improves Synapse Recovery in an In Vitro Model of Postanoxic Encephalopathy

**DOI:** 10.1007/s12035-015-9502-x

**Published:** 2015-11-06

**Authors:** Irina I. Stoyanova, Jeannette Hofmeijer, Michel J. A. M. van Putten, Joost le Feber

**Affiliations:** 1 Department of Clinical Neurophysiology, Faculty of Science and Technology, University of Twente, Building Carré 3714, P.O. Box 217, 7500 AE Enschede, The Netherlands; 2 Department of Biomedical Signals and Systems, EWI, University of Twente, Enschede, The Netherlands; 3 Department of Neurology, Rijnstate Hospital, Arnhem, The Netherlands; 4 Department of Clinical Neurophysiology, Medisch Spectrum Twente, Enschede, The Netherlands

**Keywords:** Brain hypoxia, Postanoxic encephalopathy, Synapse density, Ghrelin

## Abstract

Comatose patients after cardiac arrest have a poor prognosis. Approximately half never awakes as a result of severe diffuse postanoxic encephalopathy. Several neuroprotective agents have been tested, however without significant effect. In the present study, we used cultured neuronal networks as a model system to study the general synaptic damage caused by temporary severe hypoxia and the possibility to restrict it by ghrelin treatment. Briefly, we applied hypoxia (pO_2_ lowered from 150 to 20 mmHg) during 6 h in 55 cultures. Three hours after restoration of normoxia, half of the cultures were treated with ghrelin for 24 h, while the other, non-supplemented, were used as a control. All cultures were processed immunocytochemically for detection of the synaptic marker synaptophysin. We observed that hypoxia led to drastic decline of the number of synapses, followed by some recovery after return to normoxia, but still below the prehypoxic level. Additionally, synaptic vulnerability was selective: large- and small-sized neurons were more susceptible to synaptic damage than the medium-sized ones. Ghrelin treatment significantly increased the synapse density, as compared with the non-treated controls or with the prehypoxic period. The effect was detected in all neuronal subtypes. In conclusion, exogenous ghrelin has a robust impact on the recovery of cortical synapses after hypoxia. It raises the possibility that ghrelin or its analogs may have a therapeutic potential for treatment of postanoxic encephalopathy.

## Introduction

Comatose patients after cardiac arrest have a poor prognosis. Approximately half never awakes as a result of severe diffuse postanoxic encephalopathy [1]. Several neuroprotective agents such as barbiturates [2], calcium channel blockers [3, 4], magnesium [5], and diazepam [6] have been tested, however without significant effect. The only treatment of proven benefit is mild therapeutic hypothermia [7, 8], although the gain of this treatment has become uncertain since the recent Targeted Temperature Management (TTM) trial [9]. An important rationale behind all neuroprotective strategies, including hypothermia, has been prevention of secondary damage by inhibition of activation. This should preserve remaining energy levels in order to maintain membrane voltage and basic cellular processes and prevent excitotoxicity [10]. In acute ischemic stroke, where part of the same mechanisms play a role, many modalities have been tested based on the same argumentation [11]. Neither in postanoxic coma nor in ischemic stroke were any of these beneficial in clinical trials.

The initial encephalopathy after cardiac arrest results from a global decrease of cerebral perfusion to a level that is insufficient to meet the brain’s high metabolic demands [12]. Classically, the resulting ATP depletion is associated with failure of transmembrane ion pumps, depolarization, cell swelling, and cell death [13]. However, in imaging studies, sings of cell swelling and necrosis were only moderately associated with severity of encephalopathy and even absent in almost half of patients, despite lasting severe encephalopathy and a poor outcome [14]. Other pathophysiological mechanisms leading to brain malfunctioning include local acidosis, free radical formation, and nitric oxide production. Functional changes are caused by damage to mitochondria, cytoskeleton, and glutamate receptors [15, 16].

In animal models of acute ischemic stroke, synaptic failure through impaired transmitter release was an early consequence of ischemia and led to irreversible cerebral network damage in the absence of cell swelling or necrosis [17]. Synaptic transmission disturbances cause a lack of postsynaptic activation and consequently decreased network activity [18]. Since long-term suppression of activity leads to permanent damage [19, 20] and lack of brain activity during more than 24 h is strongly associated with absence of recovery from postanoxic coma [21, 22], it is argued that mild excitation such as caused by ghrelin, instead of inhibition, may be of benefit.

Previously, we have shown improvements of network connectivity along with an increased number of synapses, resulting from chronic stimulation of dissociated cortical neurons with ghrelin [23, 24]. Ghrelin is multifunctional 28-aminoacid hormone and a neuropeptide originally not only found in the rat stomach [25], but also identified in the hypothalamus, to a lesser extent in the hippocampus and brain cortex [26–28], as well as in the adipose tissue [29]. Ghrelin gene encodes a precursor which is cleaved to produce first unacylated ghrelin (des-acyl ghrelin (DAG)) further transformed into acyl ghrelin (AG, also referred to as ghrelin) by esterification of the serine-3 residue with *n*-octanoic acid [30]. AG binds to the growth hormone secretagogue receptor 1a isoform (GHSR1) [25], which is expressed in the cerebral cortex in vivo [27], as well as in dissociated cortical neurons [23], and is responsible for some of the central and peripheral effects of ghrelin [31]. As recently reported, ghrelin enhances synaptic plasticity [26], memory consolidation [32], and adult neurogenesis [33, 34]. Here, we show that ghrelin improves synaptic recovery in an in vitro model of postanoxic encephalopathy consisting of cultured neuronal networks exposed to severe hypoxia.

## Material and Methods

**Fig. 1:**
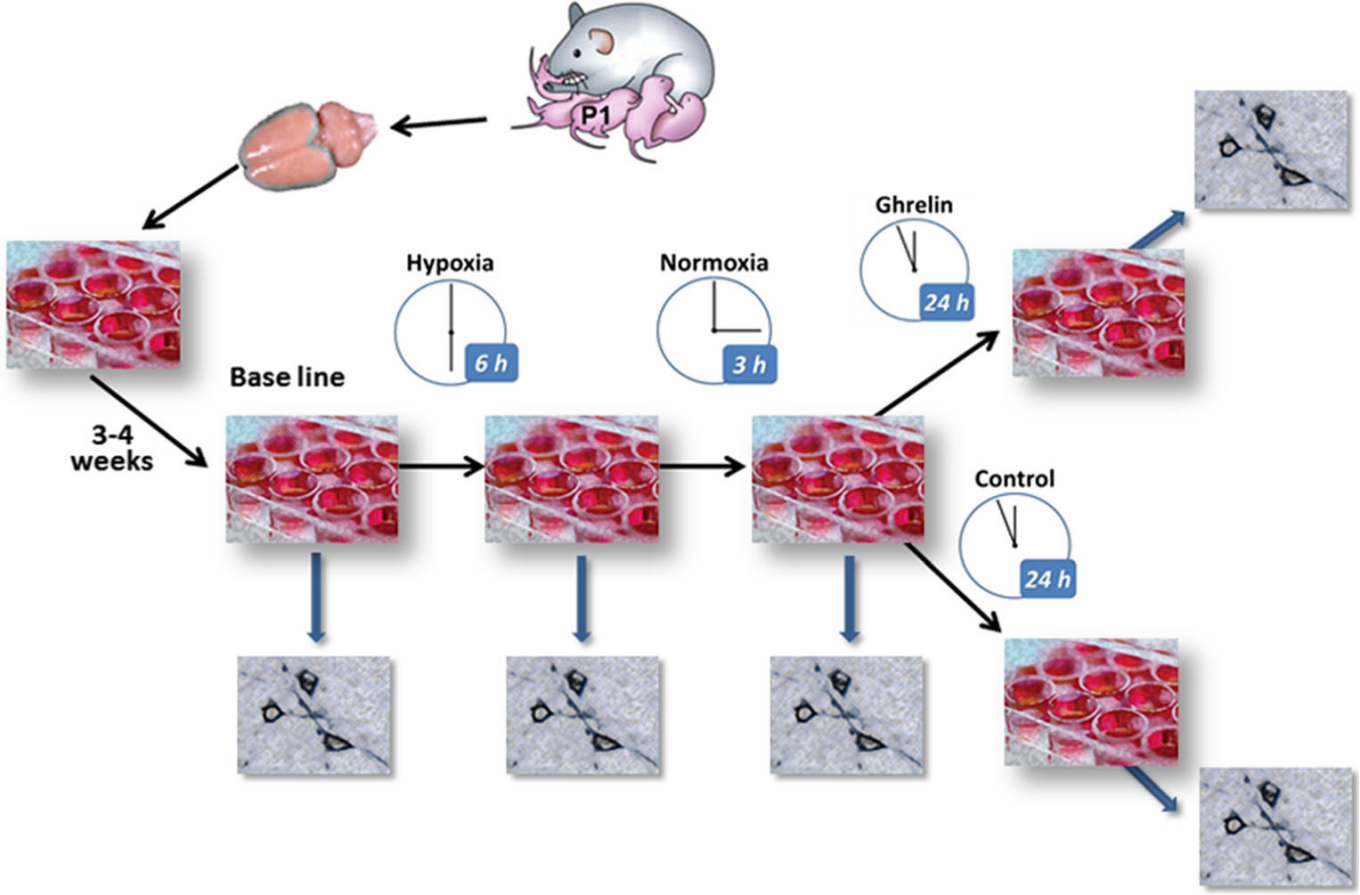
Scheme of the experimental steps: dissociation of cortical neurons and cell culturing; maturation of the networks for 3–4 weeks; experimental hypoxia for 6 h, followed by 3 h of recovery at normal oxygen supply, thereafter ghrelin treatment for 24 h. Control cultures were kept for 24 h in plane medium, not supplemented with ghrelin; immunostaining for detection of synaptophysin at the end of each experimental phase

We used an in vitro model of postanoxic encephalopathy consisting of cultured neuronal networks exposed to severe hypoxia for 6 h, followed by a recovery period with normoxia during 3 h. Thereafter, half of the cultures were supplemented with ghrelin for 24 h, while the other half were not manipulated pharmacologically and used as a control. Before induction of hypoxia and after each experimental phase, part of the neuronal cultures were fixed and processed for detection and quantitative evaluation of the synaptic marker synaptophysin (SPh). The study design is illustrated in Fig. 1.

### Dissociated Cell Cultures

Cell cultures were obtained from Wistar rat pups, from eight plating procedures, five pups (from the same mother) per plating. The animals were anesthetized with isoflurane and decapitated. The brains were removed and placed in RPMI medium (developed at Roswell Park Memorial Institute, hence the acronym RPMI). After removal of the meninges, cortical cells were dissociated and collected in chemically defined R12 culture medium [35] with trypsin for chemical dissociation. Thereafter, 150 μl of soybean trypsin inhibitor and 125 μl of DNAse I (20,000 units, Life Technology) were added, followed by trituration for mechanical dissociation of the neurons. The suspension was centrifuged at 1200 rpm for 5 min. Cells were plated on glass coverslips at a density of approximately 3000 cells/mm^2^. The glass coverslips were precoated with 20 mg/ml polyethyleneimine (Fluka, Buchs, Switzerland) to enhance cell adhesion. Cells were allowed to attach for 2 h at 37 °C and 5 % CO_2_ in air and thereafter kept in R12 medium, optimized with 50 ng/ml nerve growth factor (Invitrogen, Carlsbad, CA). The medium was serum-free to suppress glial cell proliferation, keeping their concentration lower than 5 % [35]. The medium was renewed every 2–3 days. Cultures were stored in an incubator under standard conditions of 36 °C, 100 % humidity, and 5 % CO_2_ for a period of 3–4 weeks, till the neurons established mature synaptic contacts. All animal experiments were conducted according to Dutch law (as stated in the “Wet op de dierproeven”) and approved by the Utrecht Animal Use Committee (DEC).

### Induction of Hypoxia and Pharmacological Manipulation

For induction of hypoxia, the well plates with neuronal cultures were placed under a Plexiglass hood (volume ~5 L). Temperature, humidity, and CO_2_ level of the environment were maintained as in the incubator. Two mass flow controllers were used to mix air and N_2_ and to produce a total flow of 4.7 L/min under the hood. Under physiological conditions, the gas mixture consisted of ambient air with 5 % CO_2_. For induction of hypoxia, 90 % of the air was replaced by N_2_ during 6 h, lowering the partial oxygen pressure (pO_2_) in the bath from ≈150 to ≈20 mm Hg. Restoration of normoxia followed thereafter. To obtain an estimate of the course of the oxygen concentration in the culture medium, pO_2_ was measured using an optical oxygen sensor (PHOSPOR, Ocean Optics), which was inserted into the wells, near the neurons.

Three hours after restoration of normoxia, half of the remaining cultures were supplemented with ghrelin (Abcam, Cambridge, UK) for 24 h, at a final concentration of 1 μM, as described elsewhere [24, 26, 27, 36, 37]. The other half of the cultures were kept in a plain medium, also for 24 h, and used as control.

### Immunohistochemistry

SPh staining was done on 55 cultures, equally distributed over the experimental phases (*baseline*, *6*-*h hypoxia*, *3*-*h normoxia*, *24 h Ghr*, or *24 h Ctrl*). Cultures were fixed in 4 % paraformaldehyde in 0.1 M PBS, pH 7.4, and processed immunocytochemically with the avidin-biotin-horseradish peroxidase (ABC) method [38] to detect the synaptic marker. Briefly, hydrogen peroxide (0.3 % in absolute methanol for 30 min) was used to inactivate endogenous peroxidase. Appropriate washes in PBS followed this and subsequent treatments. Incubation in primary antibody mouse anti-SPh IgG (Abcam, Cambridge, UK, dilution 1:1000) lasted for 20 h at room temperature, followed by 2 h in biotinilated donkey anti-mouse IgG (1:500; Jackson ImmunoResearch, West) and 1 h in ABC (1:500; Vector Labs, Burlingame, CA, USA). Following rinsing, peroxidase activity was visualized with 2.4 % SG substrate kit for peroxidase (Vector) in PBS for 5 min, at room temperature. Finally, the cultures were dehydrated in a graded series of alcohols, cleared in xylene, and coverslipped with Entellan (Merck, Darmstadt, Germany).

The immunoreactivity was readily discernible at the light microscopic level by the presence of dark-gray granules of immunoreactive (IR) product. Neuronal structures were considered to be immunopositive when their staining was clearly stronger than that in the background. Negative controls included incubation after antigen-antibody preabsorption with the native antigen, at 4 °C for 24 h, or replacement of the primary antibody with non-immune serum at the same concentration.

### Photomicrograph Production and Data Analysis

After staining, micrographs were generated at ×60 using a Nikon DS-F*i*1 digital camera linked to a Nikon Eclipse 50*i* microscope. All digital images were matched for brightness in Adobe Photoshop 7.0. For quantification of the synaptic marker expression, we counted the number of granules of reaction product after SPh staining. Counting was done blinded to treatment allocation. We obtained estimates of the neuronal size, the number of SPh-IR granules, and the size of the area in which they were counted by means of Nikon NIS-Elements software. To avoid bias due to the small diameter of the neurites and differences in the cell density across the cultures, we restricted this analysis to the area of the perikarya and the initial part of the arborizations.

To guarantee inclusion of neurons of various sizes in our analysis, we defined small-sized (surface of the perikaryon <100 μm^2^), medium-sized (cell 100–200 μm^2^), and large-sized neurons (cells >200 μm^2^) [39–41]. Additionally, we qualitatively graded the overall density of immunostaining of neurons into two categories, high and low, following the procedure described by Ljungdahl et al. [42]. Ten neurons from each category (low and high IR) were examined in each neuronal group (large, medium, or small sized) under each condition (baseline, 6-h hypoxia, 3-h normoxia, 24 h Ghr, or 24 h Ctrl). Thus, 60 neurons per condition were evaluated, obtained from 9 to 12 cultures per condition. SPh-IR granule density was calculated and presented as mean ± standard deviation (SD). Student’s *t* tests and a non-parametric Kruskal-Wallis test with post hoc Tukey HSD were applied to assess statistical significance of differences in SPh expression between the various groups of neurons and conditions. *P* values <0.05 were considered statistically significant.

## Results

**Fig. 2:**
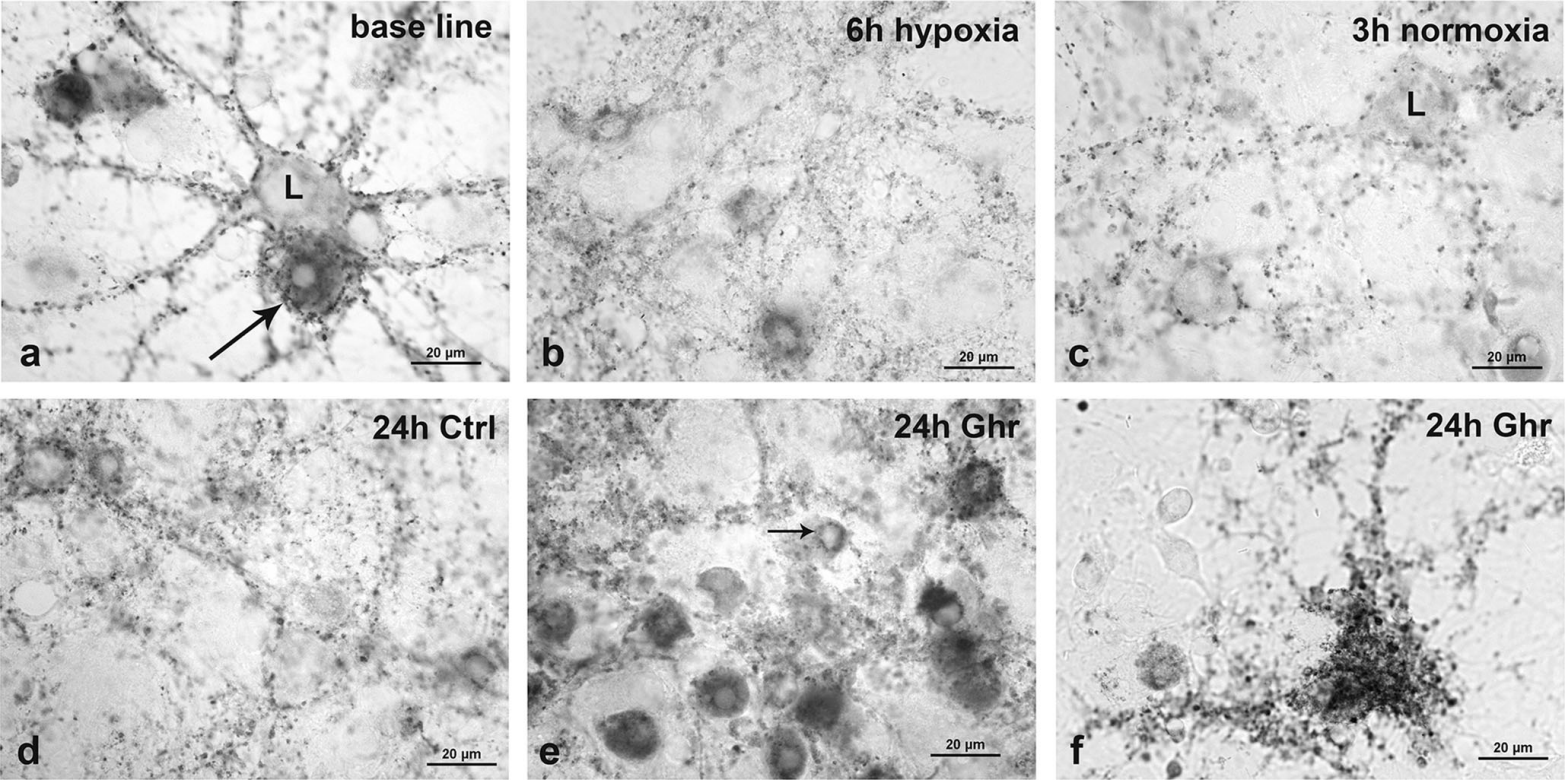
Microphotographs illustrating SPh-IR in different neuronal subtypes under each experimental condition. **a** Baseline, large (*L*)- and small-sized neurons with different SPh density. The *long arrow* is pointing at a large neuron with high density of IR granules. **b** Neurons with low SPh density after 6 h of hypoxia. **c** Neurons of different sizes after 3-h restoration of oxygen supply. *L* indicates large-sized neuron. **d** Control culture after 24-h recovery in plain medium. **e** Multiple medium-sized neurons after 24-h recovery in culture supplemented with ghrelin. Most of them show high SPh density. *Thin arrow* points at a small-sized neuron with low SPh density. **f** Twenty-four-hour ghrelin treatment, giant pyramidal neuron expressing high SPh density. *Scale bars* (**a**–**f**) 20 μm

Granules of SPh-IR product were localized on the neuronal somata and along the neurites. Their density varied according to experimental condition and according to neuronal size. Typical examples of SPh-IR neurons from each condition group are shown in Fig. 2.

### SPh Density According to Experimental Condition

Hypoxia significantly reduced SPh density (0.28 ± 0.06 granules/μm^2^) compared with baseline (0.34 ± 0.1 granules/μm^2^, *p* = 0.0002). The posthypoxic values did not return to the baseline levels, neither 3 h after restoration of normoxia (0.27 ± 0.07 granules/μm^2^) nor 24 h later (0.28 ± 0.15 granules/μm^2^). However, after ghrelin treatment at 3 h, SPh density at 24 h (0.48 ± 0.1 granules/μm^2^) was statistically significant higher than in the control group (0.28 ± 0.15 granules/μm^2^, *p* ≪ 0.001) and at baseline (0.34 ± 0.1 granules/μm^2^, *p* ≪ 0.001). The quantitative data are presented in Table 1.

**Table 1:** Density of SPh granules under different experimental conditions

Condition	Number of cultures evaluated	Number of neurons evaluated	SPh density (granules/μm^2^)	SD
Baseline	11	60	0.34	±0.10
6-h hypoxia	9	60	0.28 (**p = 0.0002)	±0.06
3-h Normoxia	12	60	0.27 (**p = 6.5.10^−5^)	±0.07
24 h Control	11	60	0.28 (*p = 0.0134)	±0.15
24 h Ghr	12	60	0.48 (**p = 2.10^−13^)	±0.10

Asterisks indicate significant change from baseline: *p < 0.05; **p < 0.001

### SPh Density According to Neuronal Size

Synapse density varied according to neuronal size. At baseline, densities were 0.38 ± 0.08, 0.30 ± 0.06, and 0.40 ± 0.12 granules/μm^2^ for large-, medium-, and small-sized neurons. Hypoxia significantly downregulated the number of synaptic contacts of the large neurons (0.29 ± 0.07 granules/μm^2^, *p* = 0.0004). This was irreversible: the density continued to decrease after restoration of normoxia (0.24 ± 0.07 granules/μm^2^ after 3 h and 0.23 ± 0.14 granules/μm^2^ 24 h later). Unlike in the large neurons, once reduced by hypoxia, SPh density of small neurons (0.28 ± 0.06 granule/μm^2^) remained unchanged during the posthypoxic periods (0.27 ± 0.05 granules/μm^2^ (3-h restoration) and 24 h later (0.31 ± 0.16 granules/μm^2^ in 24 h Ctrl). Synapse density of medium-sized neurons did not show sensitivity to hypoxia. It did not change by exposure to hypoxia (0.27 ± 0.06 granules/μm^2^) and remained unchanged after normoxia restoration (0.31 ± 0.05 granules/μm^2^ at 3 h and 0.28 ± 0.09 granules/μm^2^ 24 h later). Ghrelin significantly increased SPh density in the three neuronal subtypes. Treatment effect was largest in the small-sized neurons (Table 2).

**Table 2:** SPh density in different neuronal types under all experimental conditions

Neuronal subtype	Mean SPh density (granules/μm^2^) ± SD				
	Baseline	6-h hypoxia	3-h normoxia	24 h Ctrl	24 h Ghr
Large	0.38 ± 0.08	0.29 ± 0.07 (**p = 4.10^−4^)	0.24 ± 0.07 (**p = 1.3.10^−6^)	0.23 ± 0.14 (**p = 2.8.10^−4^)	0.45 ± 0.08 (*p = 0.01)
Medium	0.30 ± 0.06	0.27 ± 0.06	0.31 ± 0.05	0.28 ± 0.09	0.48 ± 0.09 (**p = 1.1.10^−8^)
Small	0.40 ± 0.12	0.28 ± 0.06 (**p = 3.8.10^−4^)	0.27 ± 0.05 (**p = 1.4.10^−4^)	0.31 ± 0.16 (*p = 0.04)	0.52 ± 0.12 (*p = 0.003)

Asterisks indicate significant change from baseline: *p < 0.05; **p < 0.001

## Discussion

In an in vitro model of postanoxic encephalopathy consisting of cultured neurons exposed to temporary severe hypoxia, we show a significant reduction of synaptic density (from 0.34 to 0.28 granules/μm^2^, *p* = 0.0002). Additionally, we demonstrate that treatment with ghrelin initiates recovery (from 0.28 to 0.48 granules/μm^2^, *p* ≪ 0.001) and leads to complete restoration of the synaptic density in all subtypes of neurons according to their size, with most prominent effect on the medium-sized ones (from 0.28 to 0.48 granules/μm^2^, *p* ≪ 0.001).

Our results are in line with previous in vitro and in vivo studies, showing that hypoxia or ischemia affects synaptic transmission before disruption of ion gradients across the plasma membrane [43–46]. Several causes for presynaptic and postsynaptic ischemic failure have been proposed [17]. The initial disturbances are probably located presynaptically rather than postsynaptically, with impaired transmitter release [17], decreased presynaptic dense projections [47], and isolated loss of presynaptic buttons [48]. Failure of synaptic transmission has been proposed to account for electric silence in the penumbra of a brain infarct [49] and has been associated with lasting network damage in the absence of depolarization in a rat model of cerebral infarction [45]. In addition, perinatal exposure to hypoxia caused early degenerative processes in existing synapses, decline in synaptogenesis, and a more than twofold reduction of synaptic density in all cortical layers of the rat, as shown by SPh immunodetection [50]. Cerebral ischemia induces interaction of the postsynaptic density (PSD-95) with neuronal nitric oxide synthase (nNOS), thereby leading to nitric oxide (NO) overproduction and neural injury [51]. Disruption of this coupling enhances neurite growth and dendritic spine formation and thus improves stroke outcome by promoting regenerative processes [52]. Further research on the effect of hypoxia on postsynaptic density and dendritic spine distribution would be clinically relevant.

Previously demonstrated selective ischemic damage affected striatum, hippocampal pyramidal neurons, cerebellar Purkinje cells, and the neocortical pyramidal cells [53–55] with relatively strong downregulation of synapse density [48]. In the cortex, we also observed selective vulnerability: large- and small-sized neurons were more susceptible to synaptic damage than medium-sized neurons. In general, neuronal morphology correlates with certain neuronal functions, and as the fundamental work of McConnell indicated, the large-sized neurons convey afferent sensory signals from subcortical regions and efferent signaling to subcortical areas, while the small-sized neurons are involved in efferent intracortical transmission [56]. Therefore, we could speculate that the primary sensory and motor systems are more vulnerable to ischemic damage. Such morphological evidence for selective vulnerability of the primary sensory and motor system was demonstrated in a model of asphyxic cardiac arrest in newborn piglets [57] and confirmed electrophysiologically in rodents [58]. Causes of selective vulnerability are still unclear; however, it is possible to be related to specific oxidative metabolism [57].

Previously, we reported that AG accelerates synaptogenesis and synaptic activity under healthy conditions [23, 24]. Supposed working mechanisms for stimulating synapse growth or recovery include activation of several signaling pathways. By activating GHSR1, ghrelin increases Ca^+2^ influx [59, 60] and intracellular Ca^+2^ mobilization [61] which results in synaptic gene nuclear factor expression [62]. Furthermore, as an excitatory neurotransmitter, ghrelin stimulates synaptic activity, which triggers additional synthesis and aggregation of neurotransmitter receptors [63] and other types of synaptic signaling molecules like agrin [64]. In the cerebral cortex, agrin is associated with excitatory but not inhibitory synapses [65]. It stimulates formation and stabilization of dendritic filopodia and consequently promotes synaptogenesis [66]. Additionally, agrin induces a switch from gap junction-mediated communication to synaptic transmission [67]. Considering that gap junctions respond to hypoxia-ischemia by excessive channel opening which spreads the injury [68, 69], it is reasonable to hypothesize a role for ghrelin in the restriction of ischemic injury and upregulation of the synaptic assembly via agrin-mediated mechanism.

Beneficial effects of ghrelin have been observed in animal models of acute ischemic stroke. In a rat model, administration of ghrelin reduces infarct volume and cell death [70] by preventing apoptosis and improving mitochondrial function [71]. Thus, in addition to the stimulating effect on the synaptic survival, ghrelin further ameliorates cellular respiration and network recovery from severe hypoxia.

We used cultured neuronal networks as a model system to study the effects of temporary severe hypoxia. An important advantage of this model system is that, unlike acute brain slices, cultures can stay alive for several days up to months. In addition, many neurons and synapses in a network can be studied simultaneously. A possible limitation is the lack of normal brain architecture, as present in acute brain slices. However, the focus of this study was on general synaptic damage and recovery, which does not require a specific brain structure. The system has been used before, but with a focus on anoxia-induced neuronal death instead of synaptic function [72].

In conclusion, the present observations support the hypothesis that exogenous ghrelin has a robust impact on the recovery of cortical synapses after hypoxia. It raises the possibility that ghrelin or its analogs may have a therapeutic potential for treatment of postanoxic encephalopathy.
